# 6-De­oxy-6-fluoro-d-galactose

**DOI:** 10.1107/S1600536810016612

**Published:** 2010-05-12

**Authors:** Sarah F. Jenkinson, Daniel Best, Ken Izumori, Francis X. Wilson, Alexander C. Weymouth-Wilson, George W. J. Fleet, Amber L. Thompson

**Affiliations:** a Department of Organic Chemistry, Chemistry Research Laboratory, University of Oxford, Mansfield Road, Oxford OX1 3TA, England; b Rare Sugar Research Centre, Kagawa University, 2393 Miki-cho, Kita-gun, Kagawa 761-0795, Japan; c Summit PLC, 91 Milton Park, Abingdon, Oxon OX14 4RY, England; d Dextra Laboratories Ltd, Science and Technology Centre, Whiteknights Road, Reading RG6 6BZ, England; e Department of Chemical Crystallography, Chemistry Research Laboratory, University of Oxford, Mansfield Road, Oxford OX1 3TA, England

## Abstract

The crystal structure unequivocally confirms the relative stereochemistry of the title compound, C_6_H_11_FO_5_. The absolute stereochemistry was determined by the use of d-galactose as the starting material. The compound exists as a three-dimensional O—H⋯O hydrogen-bonded network with each mol­ecule acting as a donor and acceptor for four hydrogen bonds.

## Related literature

For literature relating to the biotechnological inter­conversion of carbohydrates (Izumoring), see: Granström *et al.* (2004 ▶); Izumori (2006 ▶); Jones *et al.* (2008 ▶); Rao *et al.* (2009 ▶); Jenkinson *et al.* (2009 ▶); Gullapalli *et al.* (2010 ▶). For literature relating to fluoro­sugars, see: Cobb *et al.* (2005 ▶); Caravano *et al.* (2009 ▶); Brackhagen *et al.* (2001 ▶); Taylor & Kent (1958 ▶).
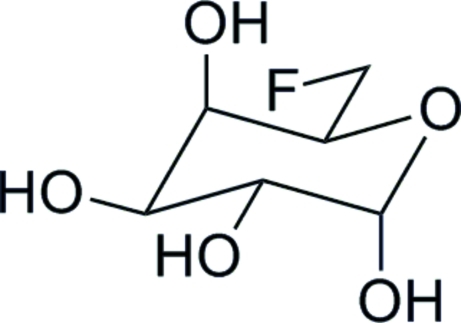


## Experimental

### 

#### Crystal data

C_6_H_11_FO_5_
*M* *_r_* = 182.15
Orthorhombic, 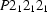

*a* = 6.7928 (3) Å
*b* = 7.5822 (3) Å
*c* = 14.1165 (6) Å
*V* = 727.06 (5) Å^3^
*Z* = 4
Mo *K*α radiation
μ = 0.16 mm^−1^
*T* = 150 K
0.25 × 0.15 × 0.15 mm

#### Data collection

Area diffractometer
Absorption correction: multi-scan (*DENZO*/*SCALEPACK*; Otwinowski & Minor, 1997 ▶) *T*_min_ = 0.88, *T*_max_ = 0.98
6912 measured reflections
978 independent reflections
855 reflections with *I* > 2σ(*I*)
*R*_int_ = 0.082

#### Refinement

*R*[*F* ^2^ > 2σ(*F* ^2^)] = 0.048
*wR*(*F* ^2^) = 0.119
*S* = 1.00
978 reflections
109 parameters
H-atom parameters constrained
Δρ_max_ = 0.39 e Å^−3^
Δρ_min_ = −0.33 e Å^−3^

### 

Data collection: *COLLECT* (Nonius, 2001 ▶); cell refinement: *DENZO*/*SCALEPACK* (Otwinowski & Minor, 1997 ▶); data reduction: *DENZO*/*SCALEPACK*; program(s) used to solve structure: *SIR92* (Altomare *et al.*, 1994 ▶); program(s) used to refine structure: *CRYSTALS* (Betteridge *et al.*, 2003 ▶); molecular graphics: *CAMERON* (Watkin *et al.*, 1996 ▶); software used to prepare material for publication: *CRYSTALS*.

## Supplementary Material

Crystal structure: contains datablocks global, I. DOI: 10.1107/S1600536810016612/lh5035sup1.cif

Structure factors: contains datablocks I. DOI: 10.1107/S1600536810016612/lh5035Isup2.hkl

Additional supplementary materials:  crystallographic information; 3D view; checkCIF report

## Figures and Tables

**Table 1:** Hydrogen-bond geometry (Å, °)

*D*—H⋯*A*	*D*—H	H⋯*A*	*D*⋯*A*	*D*—H⋯*A*
O12—H121⋯O8^i^	0.82	1.95	2.769 (4)	177
O11—H111⋯O12^ii^	0.84	1.96	2.781 (4)	168
O6—H61⋯O4^iii^	0.84	1.91	2.747 (4)	174
O8—H81⋯O6^i^	0.82	1.93	2.739 (4)	169

Symmetry codes: (i) 
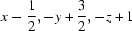
; (ii) 
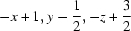
; (iii) 
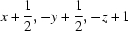
.

**Fig. 1.**
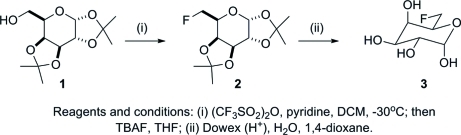
Synthetic Scheme.

**Fig. 2.**
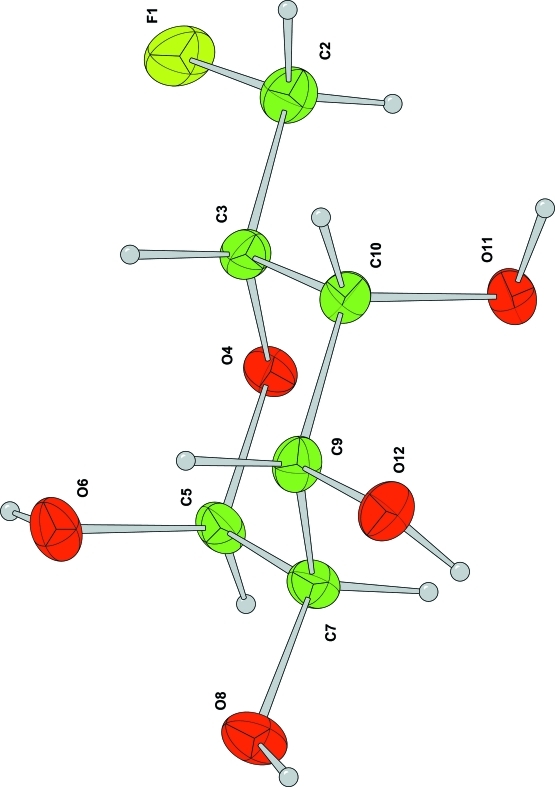
The title compound with displacement ellipsoids drawn at the 50% probability level. H atoms are shown as spheres of arbitrary radius.

**Fig. 3.**
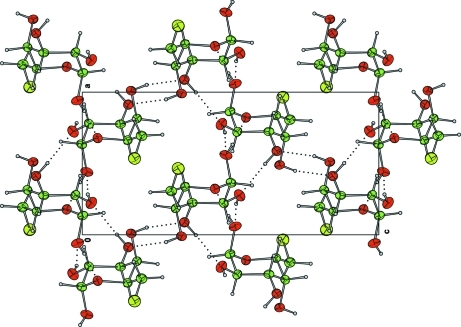
Packing diagram of the title compound projected along the b-axis. Hydrogen bonds are shown by dotted lines.

**Fig. 4.**
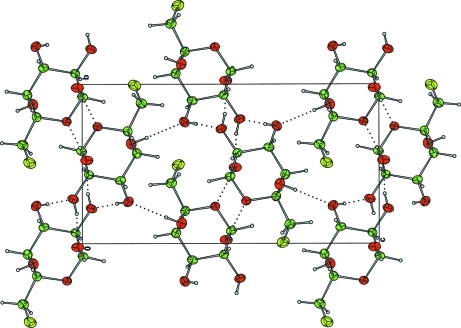
Packing diagram of the title compound projected along the a-axis. Hydrogen bonds are shown by dotted lines.

## supplementary crystallographic information

### Comment

Izumoring, a strategy for the biotechnological interconversion of aldoses, ketoses and alditols (Granström *et al.* 2004, Izumori 2006) allows convenient access to rare monosaccharides. Interconversions are achieved by regioselective microbial oxidation of alditols to give the corresponding ketoses, followed by enzymatic isomerisation to aldoses. Stereochemical diversity is introduced at C-2 in the keto-aldose isomerisation step and at C-3 by the epimerisation of ketoses, catalysed by D-tagatose-3-epimerase. In addition to the simple monosaccharides, this strategy is effective for the interconversion of deoxy (Gullapalli *et al.* 2010, Rao *et al.* 2009), methyl-branched (Jones *et al.* 2008) and azido (Jenkinson *et al.* 2009) sugars.

Fluorosugars have not been isolated from natural sources and consequently, in order to study metabolic processes, their passage along various biological pathways can be effectively tracked with the detection of fluorinated metabolites by ^19^F NMR (Cobb *et al.* 2005). The fluoro modification of sugars affects their hydrogen bonding capability and fluorosugars have been shown to resemble deoxy sugars such as fucose and rhamnose in terms of enzymatic recognition (Caravano *et al.* 2009). Application of the Izumoring strategy to fluorinated substrates would allow the bulk preparation of fluorosugars, an important and interesting class of carbohydrates.

6-Deoxy-6-fluoro-D-galactose was prepared from D-galactose diacetonide 1 (Fig. 1). Fluoride was introduced nucleophilically to give the protected fluorogalactose 2 in 68% yield as previously described for the enantiomer (Brackhagen *et al.* 2001). Dowex resin (H^+^) catalysed hydrolysis of the diacetonide gave the free 6-deoxy-6-fluoro-D-galactose 3 in 98% yield.

X-ray crystallography unequivocally confirmed the relative stereochemistry of the title compound. The absolute stereochemistry was determined by the use of D-galactose as the starting material. The compound exists as an extensively hydrogen-bonded lattice with each molecule acting as a donor and acceptor for 4 hydrogen bonds. Only classical hydrogen bonding is considered.

### Experimental

The title compound was recrystallised by vapour diffusion from a mixture of ethanol and water [m.p. 431-433 K;[α]_D_^25 ^initial: +119.8, equilibrium: +69.4 (*c* 1.12, H_2_O) {Lit. (Taylor & Kent, 1958) m.p. 433 K; [α]_D_^20^ initial: +135, equilibrium: +76.5 (*c* 0.967, H_2_O)].

### Refinement

In the absence of significant anomalous scattering, Friedel pairs were merged and the absolute configuration was assigned from the use of D-galactose as the starting material.

The H atoms were all located in a difference map, but those attached to carbon atoms were repositioned geometrically. The H atoms were initially refined with soft restraints on the bond lengths and angles to regularize their geometry (C—H in the range 0.93–0.98, O—H = 0.82 Å) and *U*_iso_(H) (in the range 1.2–1.5 times *U*_eq_ of the parent atom), after which the positions were refined with riding constraints.

### Crystal data

**Table:**

C_6_H_11_FO_5_	*F*(000) = 384
*M**_r_* = 182.15	*D*_x_ = 1.664 Mg m^−^^3^
Orthorhombic, *P*2_1_2_1_2_1_	Mo *K*α radiation, λ = 0.71073 Å
Hall symbol: P 2ac 2ab	Cell parameters from 976 reflections
*a* = 6.7928 (3) Å	θ = 5–27°
*b* = 7.5822 (3) Å	µ = 0.16 mm^−^^1^
*c* = 14.1165 (6) Å	*T* = 150 K
*V* = 727.06 (5) Å^3^	Plate, colourless
*Z* = 4	0.25 × 0.15 × 0.15 mm

### Data collection

**Table:**

Area diffractometer	855 reflections with *I* > 2σ(*I*)
graphite	*R*_int_ = 0.082
ω scans	θ_max_ = 27.4°, θ_min_ = 5.1°
Absorption correction: multi-scan (*DENZO*/*SCALEPACK*; Otwinowski & Minor, 1997)	*h* = −8→8
*T*_min_ = 0.88, *T*_max_ = 0.98	*k* = −9→9
6912 measured reflections	*l* = −18→18
978 independent reflections	

### Refinement

**Table:**

Refinement on *F*^2^	Primary atom site location: structure-invariant direct methods
Least-squares matrix: full	Hydrogen site location: inferred from neighbouring sites
*R*[*F*^2^ > 2σ(*F*^2^)] = 0.048	H-atom parameters constrained
*wR*(*F*^2^) = 0.119	Method = Modified Sheldrick *w* = 1/[σ^2^(*F*^2^) + ( 0.06*P*)^2^ + 0.71*P*], where *P* = [max(*F*_o_^2^,0) + 2*F*_c_^2^]/3
*S* = 1.00	(Δ/σ)_max_ = 0.0002
978 reflections	Δρ_max_ = 0.39 e Å^−^^3^
109 parameters	Δρ_min_ = −0.33 e Å^−^^3^
0 restraints	

### Fractional atomic coordinates and isotropic or equivalent isotropic displacement parameters (Å^2^)

**Table:**

	*x*	*y*	*z*	*U*_iso_*/*U*_eq_	
F1	0.9642 (3)	0.0074 (3)	0.67642 (15)	0.0322	
C2	0.8064 (5)	0.1203 (4)	0.7000 (2)	0.0241	
C3	0.8368 (5)	0.2957 (4)	0.6533 (2)	0.0197	
O4	0.8221 (3)	0.2665 (3)	0.55221 (14)	0.0197	
C5	0.8651 (5)	0.4205 (4)	0.4981 (2)	0.0199	
O6	1.0586 (3)	0.4776 (3)	0.51563 (17)	0.0249	
C7	0.7217 (5)	0.5681 (4)	0.5230 (2)	0.0193	
O8	0.7765 (3)	0.7197 (3)	0.46922 (16)	0.0253	
C9	0.7242 (5)	0.6033 (4)	0.6292 (2)	0.0196	
C10	0.6857 (5)	0.4332 (4)	0.6843 (2)	0.0207	
O11	0.4899 (3)	0.3738 (3)	0.66532 (15)	0.0234	
O12	0.5874 (3)	0.7379 (3)	0.65554 (15)	0.0225	
H21	0.6889	0.0662	0.6738	0.0302*	
H22	0.7979	0.1319	0.7701	0.0295*	
H31	0.9723	0.3377	0.6670	0.0229*	
H51	0.8463	0.3902	0.4278	0.0240*	
H71	0.5858	0.5333	0.5064	0.0246*	
H91	0.8539	0.6454	0.6460	0.0234*	
H101	0.7044	0.4557	0.7541	0.0250*	
H121	0.4980	0.7488	0.6170	0.0346*	
H111	0.4489	0.3361	0.7175	0.0347*	
H61	1.1320	0.3983	0.4931	0.0374*	
H81	0.7170	0.8119	0.4811	0.0389*	

### Atomic displacement parameters (Å^2^)

**Table:**

	*U*^11^	*U*^22^	*U*^33^	*U*^12^	*U*^13^	*U*^23^
F1	0.0339 (11)	0.0274 (10)	0.0352 (11)	0.0100 (9)	0.0011 (9)	0.0051 (9)
C2	0.0227 (16)	0.0222 (15)	0.0273 (15)	0.0057 (15)	0.0012 (14)	0.0016 (13)
C3	0.0187 (14)	0.0211 (14)	0.0193 (14)	0.0019 (13)	0.0003 (12)	0.0014 (12)
O4	0.0221 (11)	0.0165 (10)	0.0205 (10)	−0.0024 (9)	0.0011 (9)	0.0005 (9)
C5	0.0189 (15)	0.0168 (14)	0.0241 (14)	−0.0029 (12)	0.0029 (12)	0.0020 (12)
O6	0.0193 (12)	0.0195 (10)	0.0359 (12)	0.0006 (9)	0.0060 (10)	−0.0002 (9)
C7	0.0205 (16)	0.0171 (14)	0.0204 (14)	0.0006 (12)	0.0028 (12)	0.0018 (12)
O8	0.0307 (12)	0.0176 (10)	0.0277 (11)	0.0021 (10)	0.0077 (10)	0.0035 (9)
C9	0.0172 (15)	0.0171 (14)	0.0246 (15)	0.0033 (13)	0.0001 (12)	−0.0020 (12)
C10	0.0192 (16)	0.0228 (15)	0.0203 (14)	0.0005 (13)	−0.0016 (12)	0.0012 (12)
O11	0.0185 (11)	0.0281 (12)	0.0236 (11)	−0.0034 (10)	0.0013 (9)	0.0023 (10)
O12	0.0220 (11)	0.0230 (11)	0.0226 (10)	0.0054 (10)	−0.0005 (9)	−0.0038 (10)

### Geometric parameters (Å, °)

**Table:**

F1—C2	1.412 (4)	C7—O8	1.428 (4)
C2—C3	1.499 (4)	C7—C9	1.522 (4)
C2—H21	0.971	C7—H71	0.988
C2—H22	0.995	O8—H81	0.824
C3—O4	1.448 (3)	C9—C10	1.528 (4)
C3—C10	1.527 (4)	C9—O12	1.430 (4)
C3—H31	0.992	C9—H91	0.967
O4—C5	1.426 (4)	C10—O11	1.430 (4)
C5—O6	1.406 (4)	C10—H101	1.008
C5—C7	1.524 (4)	O11—H111	0.838
C5—H51	1.027	O12—H121	0.820
O6—H61	0.843		
			
F1—C2—C3	109.2 (3)	C5—C7—C9	110.4 (2)
F1—C2—H21	106.2	O8—C7—C9	112.3 (2)
C3—C2—H21	108.7	C5—C7—H71	110.3
F1—C2—H22	109.4	O8—C7—H71	109.4
C3—C2—H22	111.5	C9—C7—H71	106.9
H21—C2—H22	111.7	C7—O8—H81	116.5
C2—C3—O4	106.8 (2)	C7—C9—C10	110.5 (3)
C2—C3—C10	112.8 (3)	C7—C9—O12	112.0 (2)
O4—C3—C10	109.9 (2)	C10—C9—O12	111.0 (2)
C2—C3—H31	109.1	C7—C9—H91	108.1
O4—C3—H31	107.8	C10—C9—H91	108.0
C10—C3—H31	110.3	O12—C9—H91	107.0
C3—O4—C5	112.9 (2)	C9—C10—C3	108.4 (3)
O4—C5—O6	110.4 (3)	C9—C10—O11	109.2 (3)
O4—C5—C7	110.3 (2)	C3—C10—O11	110.9 (3)
O6—C5—C7	109.3 (2)	C9—C10—H101	109.5
O4—C5—H51	108.0	C3—C10—H101	108.1
O6—C5—H51	110.8	O11—C10—H101	110.7
C7—C5—H51	107.9	C10—O11—H111	104.6
C5—O6—H61	105.5	C9—O12—H121	112.3
C5—C7—O8	107.6 (2)		

### Hydrogen-bond geometry (Å, °)

**Table:**

*D*—H···*A*	*D*—H	H···*A*	*D*···*A*	*D*—H···*A*
C2—H21···O12^i^	0.97	2.60	3.318 (4)	131
C5—H51···O11^ii^	1.03	2.59	3.320 (4)	128
O12—H121···O8^iii^	0.82	1.95	2.769 (4)	177
O11—H111···O12^iv^	0.84	1.96	2.781 (4)	168
O6—H61···O4^ii^	0.84	1.91	2.747 (4)	174
O8—H81···O6^iii^	0.82	1.93	2.739 (4)	169

Symmetry codes: (i) *x*, *y*−1, *z*; (ii) *x*+1/2, −*y*+1/2, −*z*+1; (iii) *x*−1/2, −*y*+3/2, −*z*+1; (iv) −*x*+1, *y*−1/2, −*z*+3/2.

## References

[bb1] Altomare, A., Cascarano, G., Giacovazzo, C., Guagliardi, A., Burla, M. C., Polidori, G. & Camalli, M. (1994). *J. Appl. Cryst.* **27**, 435.

[bb2] Betteridge, P. W., Carruthers, J. R., Cooper, R. I., Prout, K. & Watkin, D. J. (2003). *J. Appl. Cryst.* **36**, 1487.

[bb3] Brackhagen, M., Boye, H. & Vogel, C. (2001). *J. Carbohydr. Chem.* **20**, 31–43.

[bb4] Caravano, A., Field, R. A., Percy, J. M., Rinaudo, G., Roig, R. & Singh, K. (2009). *Org. Biomol. Chem.* **7**, 996–1008. 10.1039/b815342f 19225683

[bb5] Cobb, S. L., Deng, H., Hamilton, J. T. G., McGlinchey, R. P., O’Hagan, D. & Schaffrath, C. (2005). *Bioorg. Chem.* **33**, 393–401. 10.1016/j.bioorg.2005.07.002 16165185

[bb6] Granström, T. B., Takata, G., Tokuda, M. & Izumori, K. J. (2004). *J. Biosci. Bioeng.* **97**, 89–94. 10.1016/S1389-1723(04)70173-5 16233597

[bb7] Gullapalli, P., Yoshihara, A., Morimoto, K., Rao, D., Akimitsu, K., Jenkinson, S. F., Fleet, G. W. J. & Izumori, K. (2010). *Tetrahedron Lett.* **51**, 895–898.

[bb8] Izumori, K. J. (2006). *J. Biotechnol.* **124**, 717–722. 10.1016/j.jbiotec.2006.04.016 16716430

[bb9] Jenkinson, S. F., Booth, K. V., Newberry, S., Fleet, G. W. J., Izumori, K., Morimoto, K., Nash, R. J., Jones, L., Watkin, D. J. & Thompson, A. L. (2009). *Acta Cryst.* E**65**, o1755–o1756. 10.1107/S1600536809025045 PMC2977159 21583466

[bb10] Jones, N. A., Rao, D., Yoshihara, A., Gullapalli, P., Morimoto, K., Takata, G., Hunter, S. J., Wormald, M. R., Dwek, R. A., Izumori, K. & Fleet, G. W. J. (2008). *Tetrahedron Asymmetry*, **16**, 1904–1918.

[bb11] Nonius (2001). *COLLECT* Nonius BV, Delft, The Netherlands.

[bb12] Otwinowski, Z. & Minor, W. (1997). *Methods in Enzymology*, Vol. 276, *Macromolecular Crystallography*, Part A, edited by C. W. Carter Jr & R. M. Sweet, pp. 307–326. New York: Academic Press.

[bb13] Rao, D., Best, D., Yoshihara, A., Gullapalli, P., Morimoto, K., Wormald, M. R., Wilson, F. X., Izumori, K. & Fleet, G. W. J. (2009). *Tetrahedron Lett.* **50**, 3559–3563.

[bb14] Taylor, N. F. & Kent, P. W. (1958). *J. Chem. Soc.* pp. 872–875.

[bb15] Watkin, D. J., Prout, C. K. & Pearce, L. J. (1996). *CAMERON* Chemical Crystallography Laboratory, Oxford, England.

